# Recyclable and non‐recyclable packaging films with different barrier properties: Effect of processing and storage time on quality of mashed potato and ground carrot

**DOI:** 10.1111/1750-3841.17486

**Published:** 2024-12-01

**Authors:** Nusrat Sharmin, Bjørn Tore Rotabakk, Magnhild Seim Grøvlen, Hanne Larsen, Torstein Skåra, Marit Kvalvåg Pettersen

**Affiliations:** ^1^ Department of Food Safety and Quality, Nofima – Norwegian Institute of Food Fisheries and Aquaculture Research Ås Norway; ^2^ Department of Processing Technology, Nofima – Norwegian Institute of Food Fisheries and Aquaculture Research Ås Norway

**Keywords:** barrier properties, food quality, mechanical properties, recyclable

## Abstract

The aim of this study was to evaluate if high barrier recyclable material polyethylene/ethylene vinyl alcohol (PE/EVOH) can be an alternative non‐recyclable polyamides (PA)/PE laminate and also if high barrier is required or recyclable PE material with low barrier properties is good enough to maintain the quality of thermally processed mashed potato and ground carrot. The oxygen transmission rate (OTR) of the PA/PE and PE films decreased after heat treatment, while no change was observed for PE/EVOH films. Food contact did not impact the OTR of PA/PE and PE/EVOH films, while the OTR of PE films decreased. The water vapor transmission rate (WVTR) of PA/PE and PE/EVOH films increased after heat treatment. In general, the WVTR of films increased after food contact. The tensile strength of all films was only reduced up to 3–4 weeks of food contact. After 10 weeks, the PE film showed significantly lower hue values and a larger total color difference than the two other films. Light exposure reduced the hue values and increased total color difference after 6 weeks of storage. The odor and flavor of both mashed potatoes and ground carrots were affected by light exposure. The mashed potato showed a slight reduction in freshness‐odor for all materials with storage time. For flavor, mashed potato and ground carrot showed similar trends; flavor was scored unacceptable when packaged in PE films after 6 weeks, but when packaged in PA/PE and PE/EVOH films, the flavor was still acceptable after 10 weeks of storage.

## 1 INTRODUCTION

Several different plastic materials are currently used for food packaging applications. These packaging materials typically have different degrees of permeability to small molecules such as oxygen, carbon dioxide, water vapor, and aroma, and flavor compounds. The knowledge and understanding of the permeability (barrier properties) of food packaging materials have become increasingly important as different food has different requirements to ensure longer shelf life (Trinh et al., 2023). The barrier properties can be affected by the manufacturing process and handling, as well as the packaging procedure. Thermal treatment of a packaged food can also largely affect the barrier properties of the packaging material, since temperature is a major factor that may affect the barrier characteristics (Guillard et al., 2010). Elevated temperatures typically entail increased permeability (lower barrier) and alter the crystalline/amorphous ratio which can affect the opacity of the film and change the polymer chain mobility. In addition, not only do these factors affect the barrier, but also the mechanical properties of the packaging material (Berk, 2013). Furthermore, food contact, especially with moist foods is a factor that can alter both the mechanical and barrier properties of packaging materials (Siracusa et al., 2012).

The main drivers for developments in packaging for food preservation are developments in barrier materials and demand for convenience (Abubakar Ibrahim, 2023). With several important functional properties, like resistance to abrasion, heat and chemicals, as well as high water vapor and medium gas barrier, polypropylene (PP) and polyethylene (PE) are among the most used polymers for food packaging. Polyamides (PA) have also been widely used, especially for fresh food products, and can be used in thermal processing, due to excellent thermoforming, strength, and barrier properties. For most packaged food products, the optimal packaging material must have appropriate barrier properties, processability and sealability (Massey, 2003). To address the barrier requirement of the packaging material for food, for decades, materials have been designed by combining different types of polymers into multi‐layer construction. Packaging material with high oxygen barrier is often associated with extended shelf life.

The main polymer used for oxygen barrier is ethylene vinyl alcohol (EVOH). Due to strong inter‐ and intramolecular bonding caused by the hydroxyl groups of the vinyl alcohol unit, EVOH has excellent barrier properties. But these groups are also hydrophilic. Hence, water can easily be absorbed, gradually weakening the inter‐ and intramolecular bonds and causing a decrease in barrier properties (Maes et al., 2018). Since many shelf‐stable food products are subjected to thermal treatments, which often imply harsh thermal conditions combined with the presence of water vapor, processing represents a challenging phase for both mechanical and barrier properties. Separate measurements of barrier properties of EVOH‐barrier layer materials represent neither the barrier throughout the lifespan nor the suitability as a packaging material for thermally processed foods.

European Union's (EU) Circular Economy Action Plan (CEAP) targets product design that promotes circular economy and encourages sustainable consumption. It aims to reduce waste and keep the resources in the EU economy as long as possible. A linear design with energy efficiency optimization is no longer sufficient to achieve sustainable products. This calls for the development and implementation of food packaging materials/concepts that not only allow the substitution of non‐recyclable with recyclable materials but also facilitate systems for sorting and recycling. Simultaneously, the objective to preserve food and obtain optimal shelf life should be maintained to reduce the risk of increased food waste. Overtime, several additional factors have come into play, with recyclability being of ever‐increasing importance. Although multi‐material constructions are optimal to protect and preserve the food, such complexed materials face a challenge in terms of recycling (Pauer et al., 2020; Ragaert et al., 2017). One such laminate is PA/PE, which has become a preferred material for thermally processed foods. But recyclability is imperative and non‐recyclable materials, like these, will probably be phased out. PE is recyclable, but due to its poor barrier properties, it is often combined with a barrier layer like EVOH, albeit with a maximum content of 10% to maintain recyclability (Seier et al., 2023). Polyolefins with EVOH have often been used as an alternative to non‐recyclable materials. Although many studies have been published on the structure and functional properties of PE and EVOH containing laminate (Alipour et al., 2015; Gaston et al., 2023), only limited literature is available on the effect of such materials on food quality. To the best of authors’ knowledge, no literature is available on both way interaction between the food and packaging material, where not only the effect of packaging material on the quality and shelf of food is reported but also the effect of food contact on the functional properties of the packaging material is also reported.

Although it is important to identify recyclable mono materials and laminates suitable for thermally processed foods, very limited literature is available on the effect of thermal processing on the functional properties of such material. Both analyses of material properties before and after processing and product quality characteristics related to oxidation are relevant in this respect. This study aims to compare a non‐recyclable laminate (PA/PE) with a recyclable high barrier laminate (PE/EVOH) and recyclable low barrier mono‐film material (PE), for thermally processed vegetable products, focusing on both material and product properties throughout the shelf life.

## 2 MATERIALS AND METHODS

### 2.1 Packaging material characteristics

Three different materials/laminates (Table 1) were examined in this trial. The control was a commercially available PA/PE pouch made for sous vide products (Vapak). The other pouches were made by using films of PE (low‐density polyethylene) and PE/EVOH (polyethylene/ethylene‐vinyl alcohol) and heat weld pouches with the same dimension as the PA/PE pouches.

**TABLE 1:** Packaging materials; product code name, abbreviations, product name, thickness, and producer.

Code name	Full name	Product name	Thickness (µm)	Producer
PA/PE	Polyamide/polyethylene	Vapak	20 PA/70 PE	Lietpak
PE	Low‐density polyethylene	SAC PEBD 100% recycled	100	Raja
PE/EVOH	Polyethylene/ethylene‐vinyl alcohol	Nice Eco PE XX 8	150	Wipak

Abbreviations: EVOH, ethylene vinyl alcohol; PA, polyamides; PE, polyethylene.

#### 2.1.1 Material thickness

The thickness (µm) of films of the different materials was measured by a Model 543 Film Thickness Gauge (Qualitest). Five spots on film from two different bags were measured, and the mean was calculated from the 10 measurements.

#### 2.1.2 Oxygen and water vapor transmission rate

OTR and WVTR for flat film for the three different materials were measured before packaging of food, after heating of empty bags (95°C, 100% steam for 17.5 min), and after heating and storage of food product for 6 weeks. OTR (mL O_2_ m^−2^ day^−1^) was measured at 23°C, and 50% relative humidity (RH) with a Dualperm 8001 instrument (Industrial Physics) and WVTR (g H_2_O m^−2^ day^−1^) was measured at 23°C and 85% RH using Dualperm 7002 (Industrial Physics). Four replicates were measured for all conditions.

The OTRs for whole pouches made of the different materials were determined by the ambient oxygen ingress rate (AOIR) method (Larsen et al., 2000). Empty vacuum pouches were flushed with 100% N_2_ gas (Linde Gas AS,), and O_2_ measurements were performed using a CheckMate 3 (MOCON Europe A/S (Dansensor)). For pouches with food product, the food was removed after 7 weeks of storage at 8°C, and the inside of the pouches were wiped with a paper towel before the bags were re‐sealed and flushed with 100% N_2_ gas. OTRs of bags were measured at 8°C and 70% RH on outside for three to four replicates of each sample. RH on the inside of the packages was not measured, but no water was added. OTR values for bags are given as mL O_2_ m^−2^ day^−1^ atm^−1^.

#### 2.1.3 Mechanical properties of the materials

Mashed potato and ground carrot were selected as model food for the study. The reason behind selecting mashed potato was that it is one of the most sold ready‐to‐eat foods available on the market, and ground carrot was selected as complementary product to mashed potato. Mechanical properties analyses were performed on the plastic materials before use, after heating, and after heating and food contact. Both pouches with food product and empty pouches were heat‐treated prior to the analyses. Packages with food were stored at 8°C before the food product was removed and the mechanical properties were tested. The mechanical properties of the films were measured at room temperature using an Instron 5964 equipped with a 2 kN load cell and a cross head speed of 100 mm min^−1^. The samples were prepared according to ASTM D882–18 in the form of strips. Films thickness was measured with a Japan Mitutoyo 500‐197‐20/30 200 mm/ 8′′ Digital Digimatic Vernier Caliper (0.01 mm resolution; ± 0.02 mm accuracy). The test was performed on six samples for each sample type and was analyzed using the software Bluehill 3 Version 3.72 (2010–2015 Illinoins Tools Works Inc.).

### 2.2 Product preparation, packaging, and storage

#### 2.2.1 Preparation of mashed potato and ground carrot

Fresh carrots were purchased at a local grocery store, peeled, and cut in ∼15 g pieces prior to blanching in boiling water for 3 min and cooling in ice water for 10 min. All the pieces were then ground in a food processor (Robot Coupe R‐5‐A, Robot Coupe S.A.) for 2 × 10 s. The mashed potato was prepared using instant potato mash powder with milk, according to the recipe provided by the producer (Orkla Foods Norway). In brief, 220 g of potato mash powder was added to 830 g of water. Finally, 32 g of butter was added per 1000 g of mashed potato. Portions of 100 g were packaged in pouches at 99.9% vacuum according to the design and heat‐treated in a steam cabinet (Metos) at 95°C, 100% steam for 17.5 min to achieve a pasteurization value of 
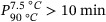
 prior to storage.

#### 2.2.2 Storage and sampling

The plastic pouches with food products were stored in darkness at 8°C and 70% RH (8.1 ± 0.5°C, 70.2 ± 4.1% RH) for up to 10 weeks. After 5 weeks of dark storage, some samples were exposed to light (LED 2500 lux) for 1 week. Food quality analyses were sampled after 3, 6, and 10 weeks of storage, and three packages of each variant were evaluated at each sampling time. Fresh samples were considered after 6 weeks of analysis.

### 2.3 Product quality

#### 2.3.1 Color

The color of the mashed potato and ground carrot products was measured through plastic materials by using a Minolta Chromameter CR‐400 (Minolta Konica Sensing Inc.) with an 8‐mm viewing port, 2° viewer angle, and illuminant D_65_. The instrument was calibrated against a white tile (L* = 97.22, a* = 0.05, and b* = 2.15). Color was measured for two spots at each package, and for three packages of each variant.

L* has a value between 0 (black) and 100 (white). Chroma C* and hue angle (*h*°) are considered to be more appropriate measures of color than CIE L*, a*, and b* (McGuire, 1992). Hence, from L*, a*, and b*, we calculated chroma (C*) and hue angle (*h*°) according to equations given in McGuire (1992) and Sant'Anna et al. (2013). Chroma expresses the vividness or saturation of a color, whereas hue angle is the degree value that corresponds to the three‐dimensional color diagram (i.e., 0° for red, 90° for yellow, 180° for green, and 270° for blue) as seen by the human eye (Sant'Anna et al., 2013). Color difference (∆E*) is a numerical comparison of a sample's color to a standard and is calculated as the Euclidean distance between two points in a three‐dimensional space defined by L*, a*, and b*(Sant'Anna et al., 2013). The color difference was calculated according to the Equation (1):

(1) $$\Delta E^{*}=\sqrt{\Delta a^{*}+\Delta b^{*}+\Delta L^{*}}.$$

The average L*, a*, and b* values from mashed potato and ground carrot measured after 1 week of storage were used as fixed standard values in calculating changes in color difference during storage.

#### 2.3.2 Appearance, odor, and flavor

Six to seven assessors evaluated the appearance of the food product, odor, and flavor. The panel was trained in using the actual method before the experiment by serving some samples and discussing and agreeing on the description and characterization. The samples were ranked on a 1–5 scale, where 5 indicates good quality (fresh with no off‐odor), 3 is deviating quality (some off‐odor), but still acceptable, and 1 indicates unacceptable food product quality (no fresh/intense off‐odor). Odor and flavor were evaluated in addition to the appearance. The products were mixed in a bag and were served to each assessor separately. Three replicates of each material variant for each food product (mashed potato and ground carrot) were randomized and given a three‐digit number and evaluated at each sampling time. Newly produced ground carrot and mashed potato were used as a known reference but were also included among the randomized samples.

#### 2.3.3 Microbiological analyses

Triplicate samples of ground carrot or mashed potato were aseptically transferred from opened bags to sterile stomacher bags and diluted 1:10 with sterile peptone water (1.0 g/L bacteriological peptone [Oxid] and 8.5 g/L NaCl) and homogenized. Quantification of total aerobic count (colony‐forming units [CFU] g^−1^) was performed on plate count agar (PCA, Merck) and incubated for 48 h at 30°C. A mechanical spiral plater (Eddy jet, IUL Instruments) was used for this purpose.

### 2.4 Statistical analysis

All statistical analyses were performed in Minitab. One‐way analysis of variance (ANOVA) was performed for the barrier properties, mechanical properties, responses bacterial counts (total viable counts), odor, flavor, appearance, and color at each sampling time. Evaluation of significant differences was performed using general linear model (GLM) ANOVA for two sets of samples as follows:

GLM (1) samples stored in different packaging materials (PA/PE, PE/EVOH, and PE) and different storage times (3, 6, and 10 weeks). The model included the main effects packaging materials (P) and storage time (T), and the interaction (P × T).
GLM (2) samples stored in different packaging materials (PA/PE, PE/EVOH, and PE) for 6 weeks in different storage conditions (dark and light). The model included the main effects packaging materials (P) and storage condition (C) (dark and light exposed), and the interaction (P × C).

## 3 RESULTS AND DISCUSSION

### 3.1 Material characteristics

#### 3.1.1 Material thickness and oxygen and water vapor transmission rate

The thickness, OTR, and WVTR of flat films and OTR of sealed bags were measured before processing, after heat treatment and after 7 weeks of contact with food (mashed potato and ground carrot, Table 2). The thickness of the PA/PE, PE/EVOH, and PE films was 79, 150, and 92 µm, respectively. For PA/PE and PE films, no significant change was observed in thickness after heat treatment and after contact with food. However, the thickness of PE/EVOH films increased by 7% and 6% after contact with mashed potato and ground carrot, respectively. As shown in Table 2, PE/EVOH films showed the lowest OTR (0.767 ± 0.113 mL m^−2^ day^−1^ atm); the OTR of PA/PE films was 26.7 ± 0.5 mL m^−2^ day^−1^ atm; however, the PE films showed the highest OTR (1596 ± 17 mL m^−2^ day^−1^ atm). EVOH is a semi‐crystalline polymer with high cohesion energy and the presence of hydrogen bonds between hydroxyl groups (Marcano et al., 2022). The presence of hydrogen bonds decreases the free volume which reduces the diffusion of oxygen through the EVOH membrane (Marcano et al., 2022). Therefore, although the thickness of PE/EVOH film was higher than both the PA/PE and PE films, the low OTR values of the PE/EVOH film were due to the EVOH barrier layer. Buntinx et al. (2014) reported the OTR of the PA/PE films measured at 25°C and 50% RH to be 26.47 and 21.3 mL m^−2^ day^−1^ atm for 166 and 293 µm film thickness, respectively, which is consistent with the value obtained in our current study. They also reported the OTR of multilayer films containing EVOH ranging between 0.48 and 1.7 mL m^−2^ day^−1^ atm which is also in accordance with the OTR values obtained for PE/EVOF films in our current study (Buntinx et al., 2014).

**TABLE 2:** Thickness, oxygen transmission rate (OTR) (23°C and 50% relative humidity [RH]) of flat film materials, water vapor transmission rate (WVTR; 23°C and 85% RH) of flat film materials, and OTR for sealed bags (8°C and 70% RH on outside).

Properties		Unprocessed—No food contact	Heat‐treated—No food contact	After contact with mashed potatoes	After contact with ground carrots
Thickness (µm)	PA/PE	79 ± 2a	81 ± 1a	83 ± 3a	82 ± 2a
	PE/EVOH	150 ± 3a	152 ± 2a	162 ± 5b	159 ± 5b
	PE	92 ± 2a	93 ± 2a	98 ± 4b	93 ± 1a
OTR (mL O_2_ m^−2^ day^−1^)	PA/PE	26.7 ± 0.50a	24.5 ± 0.70b	26.2 ± 0.50a	27.1 ± 0.50a
	PE/EVOH	0.8 ± 0.1b	0.9 ± 0.0ab	1.0 ± 0.0a	1.0 ± 0.0a
	PE	1596.8 ± 16.8c	1307.4 ± 19.5d	2412.3 ± 56.0a	1805.8 ± 67.4b
OTR sealed bags (mL O_2_ m^−2^ day^−1^ atm)	PA/PE	0.21 ± 0.00b	0.22 ± 0.01b	0.30 ± 0.00b	1.85 ± 0.47a
	PE/EVOH	0.01 ± 0.00b	0.01 ± 0.00b	0.05 ± 0.00a	0.03 ± 0.02a
	PE	20.7 ± 3.91b	19.2 ± 0.57b	25.7 ± 1.86a	17.4 ± 1.82b
WVTR (gm m^−2^ day^−1^)	PA/PE	0.6 ± 0.00c	0.7 ± 0.01bc	0.9 ± 0.02a	0.7 ± 0.00b
	PE/EVOH	0.5 ± 0.10c	0.6 ± 0.02bc	0.7 ± 0.01a	0.7 ± 0.01ab
	PE	0.8 ± 0.00c	0.8 ± 0.01 c	1.3 ± 0.05a	0.8 ± 0.01b

*Note*: Barrier properties for materials packaged with food were measured after 7 weeks of storage at 8°C and 70% RH on the outside. The values are mean ± standard deviations, and means with different letters within each material are significantly different (*p* < 0.05). The numbers with different letters are significantly different (one‐way ANOVA within each material for all measurements).

Abbreviations: EVOH, ethylene vinyl alcohol; PA, polyamides; PE, polyethylene.

After heat treatment, the OTR of PA/PE and PE films decreased by 8% and 18%, respectively. However, heat treatment did not have any significant effect on the OTR of PE/EVOH films. In addition, no significant change in the OTR was observed for the sealed bags after heat treatment. Polymer microstructure and chain mobility have a major influence on the gas permeability of the films (Blanchard et al., 2017). The high density of the crystalline phase compared to the amorphous phase in the polymer microstructure can improve the material stiffness which in turns reduces the permeability (Blanchard et al., 2017). On the contrary, increasing temperature can increase the permeability due to enhanced motion of the polymer segment and increased energy level of the permeable molecules (Mrkić et al., 2007). In our study, there was a slight increase in the OTR for PA/PE and PE films after heat treatment which could be due to the relaxation of amorphous phase after heat treatment at 90°C for 10 s resulting in higher crystallinity.

No significant change in the OTR values was observed for PA/PE and PE/EVOH films after food contact. However, for PE films, the OTR values increased by 46% and 28% after contact with mashed potato and ground carrot, respectively. No significant change in the thickness of the PE films was observed after food contact. For the sealed bags, the OTR values for PA/PE, PE, and PE/EVOH increased by 27%, 80%, and 26% after contact with mashed potato, respectively. However, no significant change in the OTR values was observed in the sealed bags after contact with ground carrots. It is well known that the impregnation of different liquid lubricants can alter the physicomechanical properties of polymers (Sędłak et al., 2017). The quantity of the liquid lubricant that can impregnate depends on its macromolecular structure and cross‐linking degree of the polymers (Sędłak et al., 2017). The increase in OTR after contact with mashed potato could be due to the impregnation of fat from the butter that was added during the preparation of mashed potato into the polymer structure.

The WVTR of PA/PE and PE/EVOH films increased by 14% and 17%, respectively, after heat treatment. In contrast, the WVTR of PE films decreased by 13% after heat treatment. These changes in WVTR could be due to the overall change in the free volume as a result of redistributed crystalline and amorphous regions in the polymer structure after heat treatment (Hirvikorpi et al., 2011). After contact with mashed potato, the WVTR of PA/PE, PE/EVOH, and PE films increased by 22%, 14%, and 46%, respectively. The WVTR of PA/PE films remained unchanged after contact with ground carrot. However, for PE/EVOH and PE films, the WVTR increased by 14% and 13% after contact with ground carrot, respectively. As explained earlier, the increase in WVTR after food contact could be due to the impregnation of liquid lubricants from food into the polymer structure.

#### 3.1.2 Mechanical properties

The effect of heat treatment and food contact on the tensile strength and elongation at break of PA/PE, PE/EVOH, and PE films (in machine direction [MD] and cross‐directional orientation [CD]) are presented in Table 3. All samples resist fairly well with the heat treatment. Statistically, the tensile strength and elongation at break values obtained showed no significant difference (*p* ˂ 0.05). The tensile strength of PE MD films was 21% lower than the PE CD samples. After heat treatment, the tensile strength of the PE MD was 26% lower than the PE CD samples. However, the elongation at break of PE MD films was around 77% higher than the PE CD films (both non‐treated and heat‐treated). The lower tensile strength and higher elongation at break values for the PE MD films could be due to the relaxation of the stresses generated during the manufacturing process (Galotto et al., 2008). In contrast, for PE/EVOH and PA/PE samples, no significant difference in the tensile strength and elongation at break was observed between the MD and CD films. It is worth mentioning that the PE/EVOH and PA/PE films are multilayer films produced by the co‐extrusion process. Therefore, no difference in the mechanical properties between MD and CD films could be due to different orientations of different layers during the manufacturing process.

**TABLE 3:** Tensile strength and elongation at break for flat film materials in machine direction (MD) and cross‐directional orientation (CD).

Properties		Unprocessed—No food contact	Heat treated—No food contact	After contact with mashed potatoes for 3 weeks	After contact with mashed potatoes for 12 weeks
	Machine direction (MD)				
Tensile strength (MPa)	PA/PE	31.84 ± 1.22a	31.41 ± 0.89a	19.64 ± 0.60c	23.92 ± 1.69b
	PE/EVOH	17.75 ± 0.58c	17.83 ± 0.80c	14.76 ± 1.82de	14.33 ± 0.62def
	PE	14.22 ± 0.31def	15.44 ± 0.43d	13.06 ± 0.66ef	12.81 ± 0.71f

Cross‐directional orientation (CD)					
	PA/PE	30.92 ± 4.02a	31.41 ± 1.11a	21.73 ± 1.84bc	22.96 ± 0.50b
	PE/EVOH	16.40 ± 5.23def	14.64 ± 0.62ef	12.61 ± 0.86ef	13.54 ± 1.56f
	PE	19.32 ± 0.93bcd	19.60 ± 0.95bcd	17.51 ± 0.45cde	19.23 ± 1.72bcd

Elongation at break (%)	Machine direction (MD)				
	PA/PE	342.03 ± 32.37e	363.04 ± 25.40de	213.54 ± 29.69 f	354.67 ± 60.07e
	PE/EVOH	396.82 ± 17.78cde	447.09 ± 16.94bc	483.26 ± 64.26b	432.92 ± 33.47bcd
	PE	604.26 ± 20.21a	603.62 ± 28.40a	509.63 ± 55.61b	637.67 ± 23.21a

Cross‐directional orientation (CD)					
	PA/PE	416.29 ± 36.52bc	422.60 ± 55.32bc	306.37 ± 66.71e	390.87 ± 24.58cd
	PE/EVOH	434.87 ± 40.92bc	476.88 ± 25.82ab	519.29 ± 48.96a	521.70 ± 45.01a
	PE	136.18 ± 33.54f	148.93 ± 45.67f	99.01 ± 4.92f	330.62 ± 47.32de

*Note*: The values are mean ± standard deviations, and means with different letters within each material are significantly different (*p *< 0.05). The numbers with different letters are significantly different (one‐way ANOVA within each material for all measurements).

Abbreviations: EVOH, ethylene vinyl alcohol; PA, polyamides; PE, polyethylene.

The tensile strength and elongation at break of all three films showed similar trends after contact with mashed potato and ground carrot. Therefore, only the change in tensile strength and elongation at break of the films after contact with mashed potato for 3 and 12 weeks is presented (Table 3). The highest reduction in the tensile strength after food contact was observed for PA/PE films. After contact with mashed potato for 3 weeks, the tensile strength of the PA/PE MD and PA/PE CD films reduced by 35% and 29%, respectively. However, with further contact with mashed potato up to 12 weeks, no additional change in the tensile strength was observed. A similar trend was also observed for PE/EVOH and PE MD films. However, the reduction in tensile strength of PE/EVOH and PE MD films after 3 weeks contact with mashed potato was much smaller compared to the PA/PE films. On the contrary, no significant change (*p*‐value) in the tensile was observed for PE CD films after food contact. The tensile strength of the PE CD and PE CD films was not affected by the food contact.

It is important to note that the PAs are hydroscopic in nature as the polar amide groups present in PAs can form hydrogen bonds with water (Razumovskii et al., 1985). These hydrogen bonds between two neighboring amide groups can alter the mobility of the molecular entities resulting in important changes in the functional properties. Touris et al. (2020) studied the effect of molecular weight and hydration on the tensile properties of polyamide 12, and they reported that water acts as a plasticizer which reduced the elastic modulus and yield stress while increased the flexibility and elongation of the material. Therefore, the reduced tensile strength of the PA/PE films observed in our study could be due to the hydroscopic nature of PAs. Tanaka et al. (2013) also reported reduction in tensile strength of carbon fiber–PA composites due to water absorption. It has been reported that with the increase in temperature, there is an increase in the water vapor permeability of polyethylene films, and as a result, the water molecules can traverse the hydrophobic polyethylene layers and reach out to the intermediate EVOH layer which is a moisture sensitive polymer (Galotto et al., 2008; López‐de‐Dicastillo et al., 2010). The water molecule can have a strong interaction with the polymer chain causing a significant impact on the cohesive energy density of EVOH, resulting in a strong impact on chain mobility and mechanical properties (Blanchard et al., 2017). Similarly, the reduction in tensile strength of PE/EVOH MD could be due to the moisture sensitive nature of EVOH.

No significant difference was observed in elongation at break for PE/EVOH films in either directions’ films after contact with mashed potato compared to the heat‐treated films. The elongation at break of PA/PE MD reduced by 41% after 3 weeks. However, with further contact with mashed potato up to 12 weeks, the elongation at break of the PA/PE MD films increased and regained the value observed for the heat‐treated samples. It has already been mentioned that the moisture content has a dramatic effect on the mechanical and physical properties of PAs. The presence of water in the amorphous phase of the PA can lead to a large decrease in both Young's modulus and stress at yield due to the mobility of macromolecular chains (Le Gac et al., 2017). Touris et al. (2020) reported that as hydration increases, the PAs become more elastic. However, the effect of water on the elasticity of the PAs is not permanent, and the material can go back to its initial stage. A similar trend of change in elongation at break after food contact was also observed for PA/PE CD and PE MD films. For PE CD films, an initial reduction in the elongation break was observed after 3/4 weeks of contact with mashed potatoes and minced carrot. However, with further contact with mashed potato up to 12 weeks, the elongation at break increased by 55% compared to the heat‐treated samples.

### 3.2 Product quality

#### 3.2.1 Effect of storage time

##### 3.2.1.1 Color

**FIGURE 1:**
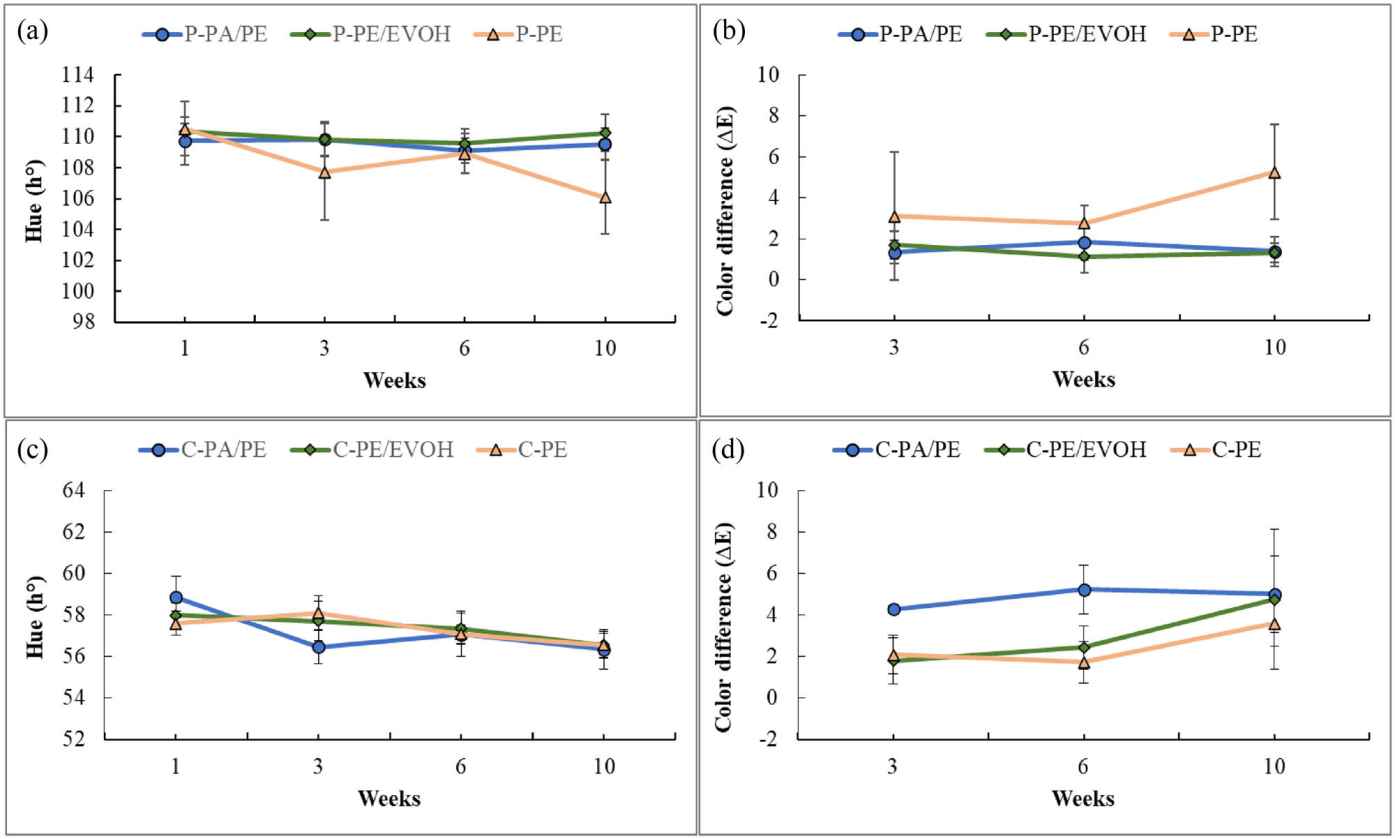
Hue for mashed potato and ground carrot (a and c) and total color difference for mashed potato and ground carrot (b and d) stored in dark for 1, 3, 6, and 10 weeks. The values are mean of six measurements, and the error bars represent the standard deviation. EVOH, ethylene vinyl alcohol; PA, polyamides; PE, polyethylene.

The color of the mashed potato and ground carrot was measured after 1, 3, 6, and 10 weeks of dark storage. Hue and color difference were the color parameters showing the largest differences between samples, especially for mashed potato (Figure 1). A complete overview of all color parameters and associated statistics are presented in Table 4 and Table S1.

**TABLE 4:** Results from one‐way analysis of variance (ANOVA) for color of mashed potato and ground carrot: Effect of packaging after 1, 3, 6, and 10 weeks of dark storage, and effect of packaging in dark and light storage after 6 weeks of storage.

Mashed potato							
						6 Weeks of storage	
		1 Week	3 Weeks	6 Weeks	10 Weeks	Darkness	Light exposure
L*	PA/PE	74.9a	74.8a	75.9a	74.8a	75.9a	73.9bc
	PE/EVOH	74.1ab	73.6a	74.3b	74.7a	74.3ab	73.2bc
	PE	73.7b	74.1a	75.7a	73.3a	75.7a	72.2c
C*	PA/PE	14.8a	14.2a	14.7a	14.0a	14.7a	9.7b
	PE/EVOH	14.2a	13.9a	14.1a	13.8a	14.1a	9.8b
	PE	14.2a	13.7a	14.5a	13.1a	14.5a	9.7b
*h*°	PA/PE	109.7a	109.8a	109.1a	109.6a	109.1ab	104.5c
	PE/EVOH	110.3a	109.8a	109.6a	110.3a	109.6a	105.9bc
	PE	110.5a	107.7a	108.9a	101.6b	108.9ab	99.8d
**∆** *E*	PA/PE		1.4a	1.8ab	1.4b	1.8c	7.5b
	PE/EVOH		1.7a	1.1b	1.3b	1.1c	6.6b
	PE		3.1a	2.8a	9.5a	2.8c	11.9a

Ground carrot							
						6 Weeks of storage	
		1 week	3 weeks	6 weeks	10 weeks	Darkness	Light exposure
L*	PA/PE	51.8ab	53.2a	52.6a	52.8a	52.6a	51.9a
	PE/EVOH	51.3b	52.0a	51.9a	53.6a	51.9a	52.0a
	PE	52.9a	52.2a	52.8a	52.1a	52.8a	52.2a
*C* *	PA/PE	50.1a	47.4a	45.3b	46.0a	45.3b	45.6b
	PE/EVOH	46.2b	47.3a	46.7ab	49.6a	46.7ab	46.4ab
	PE	49.0a	48.0a	48.7a	45.9a	48.7a	46.8ab
*h* **°**	PA/PE	58.9a	47.4a	57.1a	56.4a	57.1ab	55.9bc
	PE/EVOH	58.0b	47.3a	57.4a	56.6a	57.4a	55.8c
	PE	57.6b	48.0a	57.1a	56.6a	57.1ab	56.4abc
**∆** *E*	PA/PE		4.3a	5.3a	5.0a	5.3a	5.4a
	PE/EVOH		1.8b	2.4b	4.8a	2.4b	3.0b
	PE		2.1b	1.7b	3.6a	1.7b	2.9b

*Note*: The numbers with different letters within each storage time and color parameter are significantly different (p < 0.05).

Abbreviations: EVOH, ethylene vinyl alcohol; PA, polyamides; PE, polyethylene.

For mashed potato, the hue values and color difference were stable during the first 6 weeks of storage, and no significant differences were detected between the packaging variants. After 10 weeks of storage, mashed potato in PE film showed significantly lower hue values and larger total color differences than PA/PE and PE/EVOH, even though the PE film showed large sample variation in hue and color difference values. A large drop in hue values after 60 days of storage was also reported by Sonar et al. (2020) for pasteurized mashed potato packaged in low barrier film and stored at 5°C. A color difference of 3 < Δ*E* < 6 is considered a perceptible difference to human eyes (Sonar et al., 2020), as was noticed for the PE film samples in our trial [28].Ground carrot showed a small decrease in hue values from average 58.2–56.5 after 10 weeks storage. No significant differences were found for hue values between the packaging materials. Ground carrot in PA/PE (OTR value = 27 mL m^−2^ day^−1^ atm) had significantly higher color difference values than PE/EVOH and PE after 3 and 6 weeks, but there were no significant differences after 10 weeks of storage. The color difference was relatively stable for the PA/PE samples during the storage period, whereas a small increase from 2 to 4–5 could be noticed for PE/EVOH and PE samples. Differences in color were difficult to visually detect, in line with Zhang et al. (2016) who reported a color difference of 3 and above as visually perceptible.

The higher color difference for PA/PE is difficult to explain with respect to the OTR values. Sonar et al. (2019) found that the carrot puree stored in the low OTR pouch had significantly (*p* < 0.05) lower color differences than puree in the medium and high OTR (0.99 ± 0.05, 29.8 ± 1.38, and 80.9 ± 2.15 cm^3^ m^−2^ day^−1^, respectively) pouches during storage. However, the overall color differences in the puree in all three types of pouches were below 3, not being visually perceptible according to Zhang, Bhunia, et al. (2016).

##### 3.2.1.2 Appearance, odor, and flavor

**FIGURE 2:**
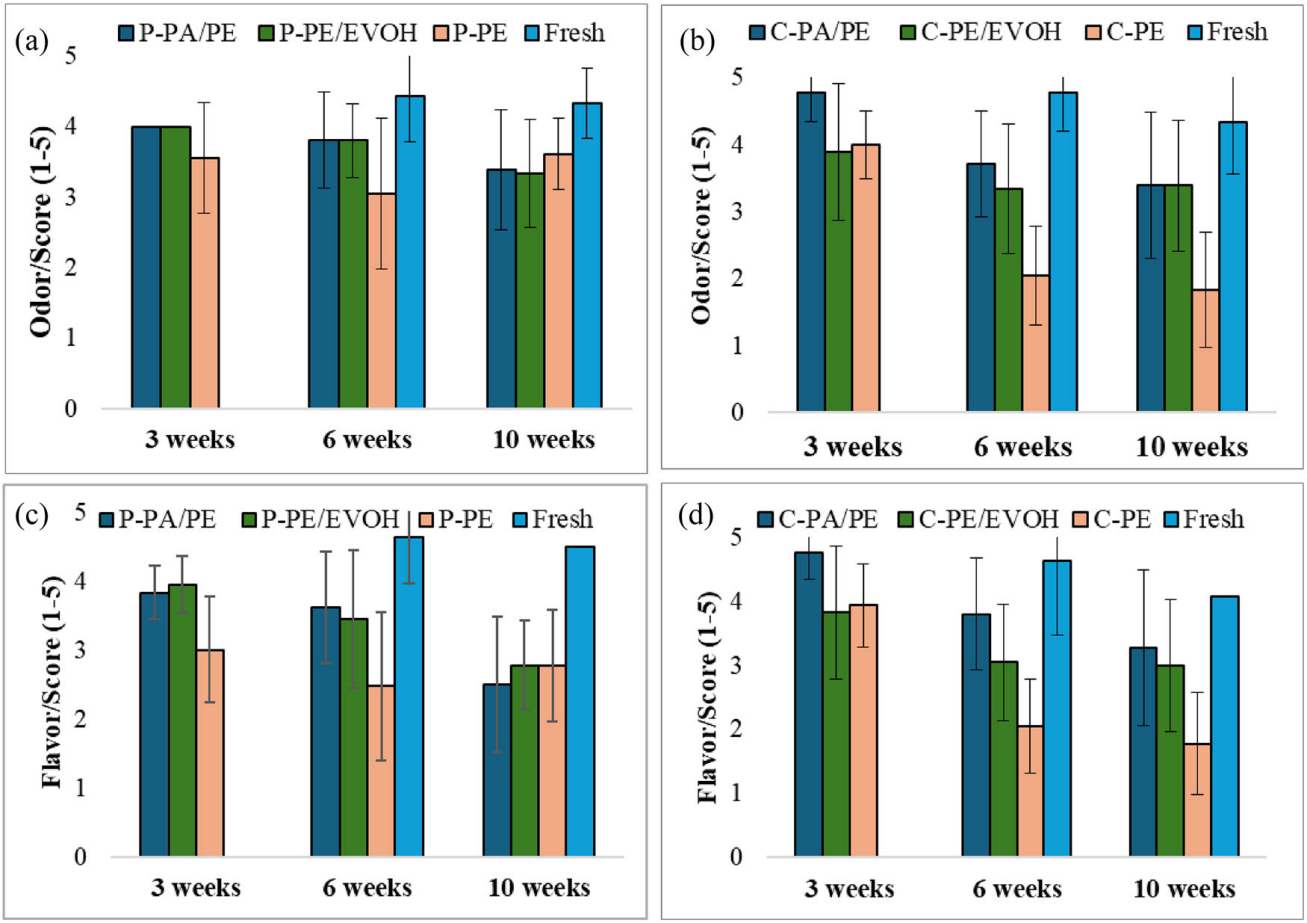
Odor and flavor of mashed potato (a–c) and ground carrot (b–d) stored in dark for 3, 6, and 10 weeks. The values are mean of four measurements, and the error bars represent the standard deviation. EVOH, ethylene vinyl alcohol; PA, polyamides; PE, polyethylene.

The effect of storage condition (dark/light exposure) on the appearance, odor, and flavor was evaluated by the lab panel. The odor of the mashed potato showed a slight reduction in freshness‐score for all materials with storage time (Figure 2a). The recyclable barrier (PE/EVOH) and non‐recyclable reference (PA/PE) both showed a score of 4 after 3 weeks of storage. On the other hand, storage in PE resulted in a slightly lower freshness‐score (3.5) than storage in materials with higher oxygen barriers. After 6 weeks, mashed potato was evaluated to score 3.0 in PE and 3.8 in PE/EVOH and PA/PE. After 10 weeks of storage, the odor was regarded similar in all packaging materials with no significant differences (Table 5).

**TABLE 5:** One‐way analysis of variance (ANOVA): Odor, flavor, and appearance in mashed potato and ground carrot.

Mashed potato						
					6 Weeks of storage	
		3 Weeks	6 Weeks	10 Weeks	Darkness	Light exposure
Odor	PA/PE	4.0a	3.81a	3.39b	3.81ab	3.38bc
	PE/EVOH	4.0a	3.80a	3.33b	3.80ab	3.05c
	PE	3.56b	3.05b	3.61b	3.05c	1.67d
	Fresh		4.43a	4.33a	4.43a	
Flavor	PA/PE	3.83a	3.62b	2.5b	3.62b	2.71cd
	PE/EVOH	3.94a	3.45b	2.78b	3.45bc	2.43d
	PE	3.0b	2.48c	2.78b	2.48d	1.19e
	Fresh		4.64a	4.50a	4.63a	
Appearance	PA/PE	5.0a	4.67a	4.35a	4.67a	2.33c
	PE/EVOH	5.0a	4.67a	4.44a	4.67a	2.83c
	PE	4.6b	4.0b	3.17b	4.0b	1.61d
	Fresh					

Ground carrot						
					6 Weeks of storage	
		3 Weeks	6 Weeks	10 Weeks	Darkness	Light exposure
Odor	PA/PE	4.78a	3.71b	3.39b	3.71b	3.76b
	PE/EVOH	3.89b	3.33b	3.39b	3.33bc	2.69cd
	PE	4.0b	2.05c	1.83c	2.05d	1.95d
	Fresh		4.79a	4.33a	4.78a	
Flavor	PA/PE	4.78a	3.81b	3.28b	3.81ab	3.52b
	PE/EVOH	3.83b	3.05c	3.0b	3.05b	2.10c
	PE	3.94b	2.05d	1.78c	2.05c	1.57c
	Fresh		4.64a	4.08a	4.64a	
Appearance	PA/PE	4.63a	4.41a	4.45a	4‐41b	4.78ab
	PE/EVOH	4.67a	4.35a	4.67a	4.35b	5.0a
	PE	4.1b	4.44a	3.68b	4.44ab	3.78c
	Fresh					

*Note*: One‐way ANOVA was performed at each sampling time. Numbers with different letters within sampling time are significantly different. Six weeks of dark/light storage (one‐way ANOVA for all samples stored for 6 weeks): Numbers with different letters with dark/light are significantly different (p < 0.05).

Abbreviations: EVOH, ethylene vinyl alcohol; PA, polyamides; PE, polyethylene.

A similar trend was observed in the flavor of the mashed potato (Figure 2c), with similar scores for PE/EVOH (score of 3.9) and PA/PE (score of 3.8) compared to a lower score for PE (score of 3.0) after 3 weeks of storage. However, the reduction in freshness flavor was more pronounced compared to the odor. Mashed potato stored in PE was regarded notably altered with a score below 3 after 6 weeks of storage. The changes in odor and flavor for potato stored in PE appear to be arbitrary—with a reduction in score from 3 to 6 weeks followed by an increase, in contrast to storage in PE/EVOH and PA/PE. According to GLM (1 = 3–10 weeks, dark storage) the main effects, material (packaging) and storage (time) as well as the interaction (P × T), significantly affected odor and flavor. However, the models were not very well fitted (*R* ^2^ adj. 28.2% and 13.6% for flavor and odor, respectively), and the packaging and storage time explained 9% and 14% of the variation, respectively, in the flavor and only 4.2% and 5.4% of the variation in odor (Table 2a).

Storage in PE/EVOH and PA/PE resulted in higher flavor scores compared to PE, and no significant differences were observed between these materials during the storage time. These changes and differences correspond well to the OTR of the materials. The most pronounced changes in flavor were from 6 to 10 weeks in the barrier materials. All samples were evaluated as less fresh with score below 3 after 10 weeks of storage, and significantly different from the fresh sample. This might indicate that 10 weeks is beyond the acceptable shelf life of this product. Although there was a slight tendency to increased microbial growth during storage, the differences were not significant (as stated in Section 3.2.2.3). Hence, no clear correlation can be stated between the flavor results and the microbial levels detected in the mashed potato. According to our knowledge, other works including evaluation of odor and flavor for similar products and process are scarce. Zhang et al. (2019) studied sensory retention in sweet potato puree stored in different multilayer materials. They reported significant changes in liking of flavor and overall acceptance for sweet potato (Zhang et al., 2019). The color and appearance of food, and especially for vegetables, are of importance for consumer acceptance at purchase reported by Peng et al. (2017).

According to Catauro and Perchonok (2012), the color tends to darken in starch‐based products, while color fading is one of the major degradations in products with high levels of carotenoid (Catauro & Perchonok, 2012). The appearance of products (inside the packages before opening) was evaluated (the figure is not shown). After 3 weeks of storage, no changes in the appearance were seen in neither PA/PE nor PE/EVOH, while they were slightly altered (score 4.6) in PE. For the potato stored in PE, the preponderant changes in appearance after 6 and 10 weeks of storage resulted in a certain reduction in appearance (score of 3.17) after 10 weeks of storage, while only minor changes were observed in PE/EVOH and PA/PE (score of 4.7 and 4.4 after 6 and 10 weeks, respectively). Others have reported increase in lightness (L*), redness (a*), and yellowness (b*) due to microwave‐assisted pasteurization systems (MAPS) for mashed potatoes (Sonar et al., 2020). In our study, mashed potato stored in PE had significantly lower score at each sampling time compared to storage in the barrier materials PA/PE and PE/EVOH. No significant differences were detected between PA/PE and PE/EVOH at any sampling time (Table 5). These results correspond well to the measured color of the mashed potato with significant lower hue values in PE compared to the materials with higher oxygen barrier, PA/PE and PE/EVOH (Figure 1). Moreover, the results are in line with the measured total color differences, with Δ*E* of 9 for mashed potato stored in PE after 10 weeks which is detectable to the human eye (Zhang, Tang et al., 2016). For the other materials, the difference was below 3.1 and probably not detectable and thus not observed by the lab panelist. Sonar et al. (2020) investigated the stability of pasteurized mashed potato and reported that the color was stable over the storage period (90 days) in all films, but with significant changes in the low barrier film (Sonar et al., 2020). Darkening has been reported by Catauro and Perchonok (2012), as the main color changes (in long term storage of canned products) for starch products. Both fading and darkening were noted in their study, while the latter was the most significant color change.

Figure 2b–d represents the odor and flavor of the ground carrot stored in PA/PE, PE/EVOH, and PE for up to 10 weeks of storage. Similar results were observed for odor and flavor with only slight alteration after 3 weeks of storage with significantly lower score in PE and PE/EVOH (score of 4/3.9 and 3.9/3.8 [odor/flavor], respectively) compared to PA/PE (score of 4.8). This is not in accordance with the barrier properties of the materials. After 6 weeks of storage, the largest reduction in score was observed for the carrot stored in PE for both odor and flavor with a score of 2.0, and thus regarded as not acceptable. The odor of ground carrot stored in PA/PE and PE/EVOH was evaluated with a score of 3.7 and 3.3, respectively. The differences in odor were not significant, whereas the flavor score was significantly lower for carrot stored in PE/EVOH (3.0) compared to PA/PE (3.8). After 10 weeks, the odor‐score of ground carrot stored in both barrier materials (PA/PE and PE/EVOH) was 3.4, while the flavor‐scores were slightly lower although not significant; 3.0 and 3.3 for PE/EVOH and PA/PE, respectively. According to GLM (1 = 3–10 weeks, dark storage), both material (packaging) and storage (time), as well as the interaction (P × T), significantly affected the odor and flavor of the minced carrot. The packaging explained 21.2% and 20.1% of the odor and flavor, respectively. Correspondingly, the storage time explained 24.1% and 26.3% of the variation (model *R* ^2^ adj. was 50.3% and 49.1% for odor and flavor, respectively; Table 2a). The effect of storage time on odor and flavor is correlated to the stated effect on microbial growth. However, the observed difference in odor and flavor as affected by the packaging materials does not correspond to findings in the microbial growth in the ground carrot.

The appearance of the carrot was evaluated (not presented in this article). Color fading is one of the major degradations in products with high levels of carotenoid (Catauro & Perchonok, 2012). The appearance score was reduced to 4.1 in PE after 3 weeks of storage and significantly lower compared to storage in the barrier materials PA/PE and PE/EVOH with no significant changes (4.9 and 5.0, respectively) (Table 5). No significant differences in appearance were observed after 6 weeks of storage with score 4.4 in all materials. After 10 weeks of storage, ground carrot stored in PE had significantly lower score (3.7) than in the barrier materials, and only minor changes were observed in PE/EVOH (score of 4.7) and PA/PE (score of 4.5) in PE/EVOH and 4.6 in PA/PE). According to color measured by Minolta ground, carrot stored in PA/PE resulted in the highest color difference values during the storage time. Similar differences were not detected by the lab panel in the evolution of the appearance; however, the evaluation was described as an overall appearance and not specifically the color (parameters).

Zhang et al. (2019) have reported almost unchanged lightness (L* value) in microwaved‐assisted thermally sterilized (MATS) sweet potato puree stored in multilayered high barrier materials for months, while an increase in yellowness (b*) (39.2–44.0) and slight reduction in redness (a*) (24.1–22.8) (Zhang et al., 2019). They also found that the color changes were significantly affected by the packaging material with different levels of OTR.

These results indicate that the barrier properties of the materials are of importance. The low barrier PE resulted in unacceptable flavor in mashed potato after 6 weeks of storage compared to 10 weeks storage in high barrier materials. The recyclable PE/EVOH had lower OTR compared to non‐recyclable PA/PE. However, storage in PE/EVOH did not result in a significantly higher score for odor, flavor, and appearance for mashed potato and ground carrot at any sampling time. However, the low barrier PE resulted in unacceptable flavor in mashed potato after 6 weeks of storage compared to 10 weeks storage in high barrier materials. Similar trends regarding the low barrier PE with not acceptable after 6 weeks were observed for ground carrot. On the contrary, the ground carrot was not regarded as unacceptable in the high barrier materials after 10 weeks of storage. This shows that different products require different barriers and protection.

##### 3.2.1.3 Microbial levels

There was no significant difference in the microbial levels between the different packaging materials for ground carrot (*p* = 0.772) or mashed potato (*p* = 0.568) (Table 6). Ground carrot had a significant (*p* = 0.002) increase from day 0 (1.99 ± 0.72 log CFU g^−1^) to week 3 (5.29 ± 1.97 log CFU g^−1^); however, there was no significant change in microbial levels over the rest of the storage period (*p* = 0.071). This contrasts with the mashed potato, where no significant differences were detected during the whole storage period (*p *= 0.458).

**TABLE 6:** One‐way analysis of variance (ANOVA): Microbial levels in mashed potato and ground carrot.

Mashed potato						
					6 Weeks of storage	
		3 Weeks	6 Weeks	10 Weeks	Darkness	Light exposure
Micro log CFU g^−1^	PA/PE	5.18	6.09a	6.01	6.09	6.06
	PE/EVOH	5.93	6.60ab	5.93	6.60	6.46
	PE	4.76	7.70b	6.06	7.70	8.09

Ground carrot						
					6 Weeks of storage	
		3 Weeks	6 Weeks	10 Weeks	Darkness	Light exposure
Micro log CFU g^−1^	PA/PE	4.98	3.31	5.61	3.31	3.01
	PE/EVOH	2.96	5.90	6.41	5.90	3.01
	PE	5.32	3.46	3.86	3.46	3.01

*Note*: One‐way ANOVA was performed at each sampling time. Numbers with different letters within sampling time are significantly different. Dark/light storage for 6 weeks (one‐way ANOVA for all samples stored for 6 weeks): Numbers with different letters with dark/light are significantly different (p < 0.05).

Abbreviations: CFU, colony‐forming unit; EVOH, ethylene vinyl alcohol; PA, polyamides; PE, polyethylene.

#### 3.2.2 Effect of storage conditions—Light exposure

##### 3.2.2.1 Color

After 5 weeks of storage, samples were exposed to light for 1 week. The effects of storage conditions (dark/light exposure) were studied for these samples.

**FIGURE 3:**
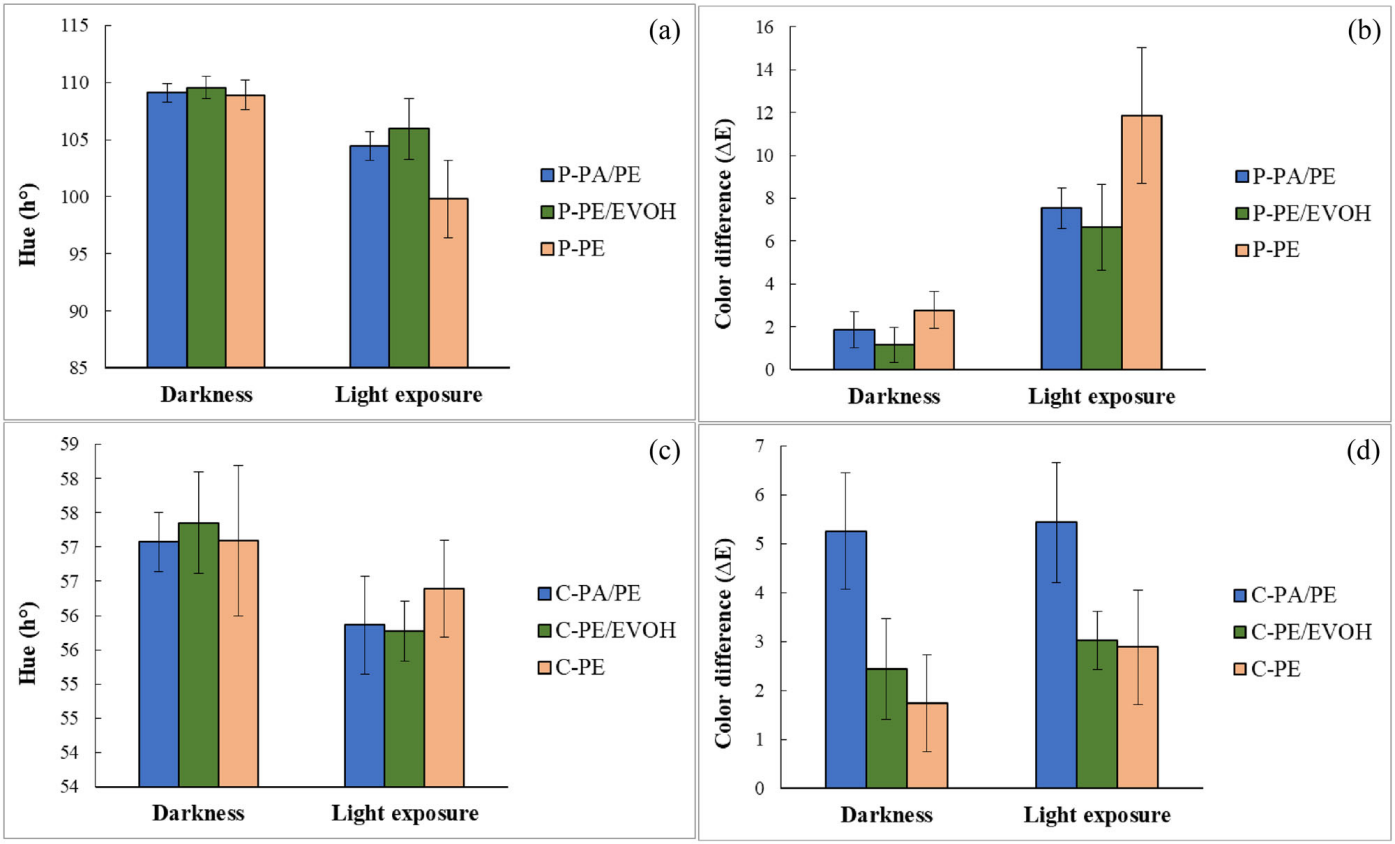
Hue for mashed potato and ground carrot (a and c) and total color difference for mashed potato and ground carrot (b and d) stored for 5 weeks in darkness + 1 week under light exposure. The values are mean of four measurements, and the error bars represent the standard deviation. EVOH, ethylene vinyl alcohol; PA, polyamides; PE, polyethylene.

Figure 3 presents the color (hue and total color difference) of the mashed potato and ground carrot stored in darkness for 5 weeks followed by 1 week storage under light exposure. The statistic parameters are presented in Table 4 and Table S1.

Light decreased hue values and increased the total color difference for mashed potato for all packaging materials after 6 weeks of storage. PE showed significantly lower hue values and higher total color differences, indicating that the light in combination with high OTR and hence higher O_2_ access led to higher oxidation rate of the pigments in mashed potato. Pettersen et al. (2022) detected a significant color change during 1 week of light exposure for sous‐vide whole potatoes packaged in materials with an OTR above 0.5 mL O_2_ pkg^−1^ day^−1^ (OTR measured at 4°C/90% RH with 0% RH on inside). The potatoes developed a grayish color with a large drop in b* values.

The orange color of the carrot is primarily due to its β‐carotene content, and β‐carotene is very sensitive to heat, oxygen, and light (Ayvaz et al., 2012). In our trial, hue values for ground carrot were slightly lower in light compared to dark storage, but significant differences between the packaging materials were not detected. The color difference, meaning the total change in color from 1 to 6 weeks, was highest for ground carrot in the PA/PE pouches. However, there were no differences between storage in light or darkness in either this material, but not PE/EVOH, or PE. The high color difference for PA/PE was due to higher values for b* (yellowness) in week 1 compared to week 6. These findings are difficult to explain in relation to the OTR of the 3 materials. Similar trials exploring the effect of light and different packaging materials on color changes in heat‐treated carrot product have been difficult to find in the literature.

##### 3.2.2.2 Appearance, odor, and flavor

In the present study, light exposure clearly affected the odor of mashed potato stored in PE with a significant higher score 3 in dark compared to score 1.7 after light exposure, and were regarded as not acceptable (Table 5). The light resulted in a slight but not significant reduction in the odor when stored in PA/PE (3.8 in dark to 3.4 after light exposure). On the contrary, light exposure resulted in a significant lower score when stored in PE/EVOH (3.8 in dark and 3.0 after light exposure). However, both samples were regarded as acceptable.

**FIGURE 4:**
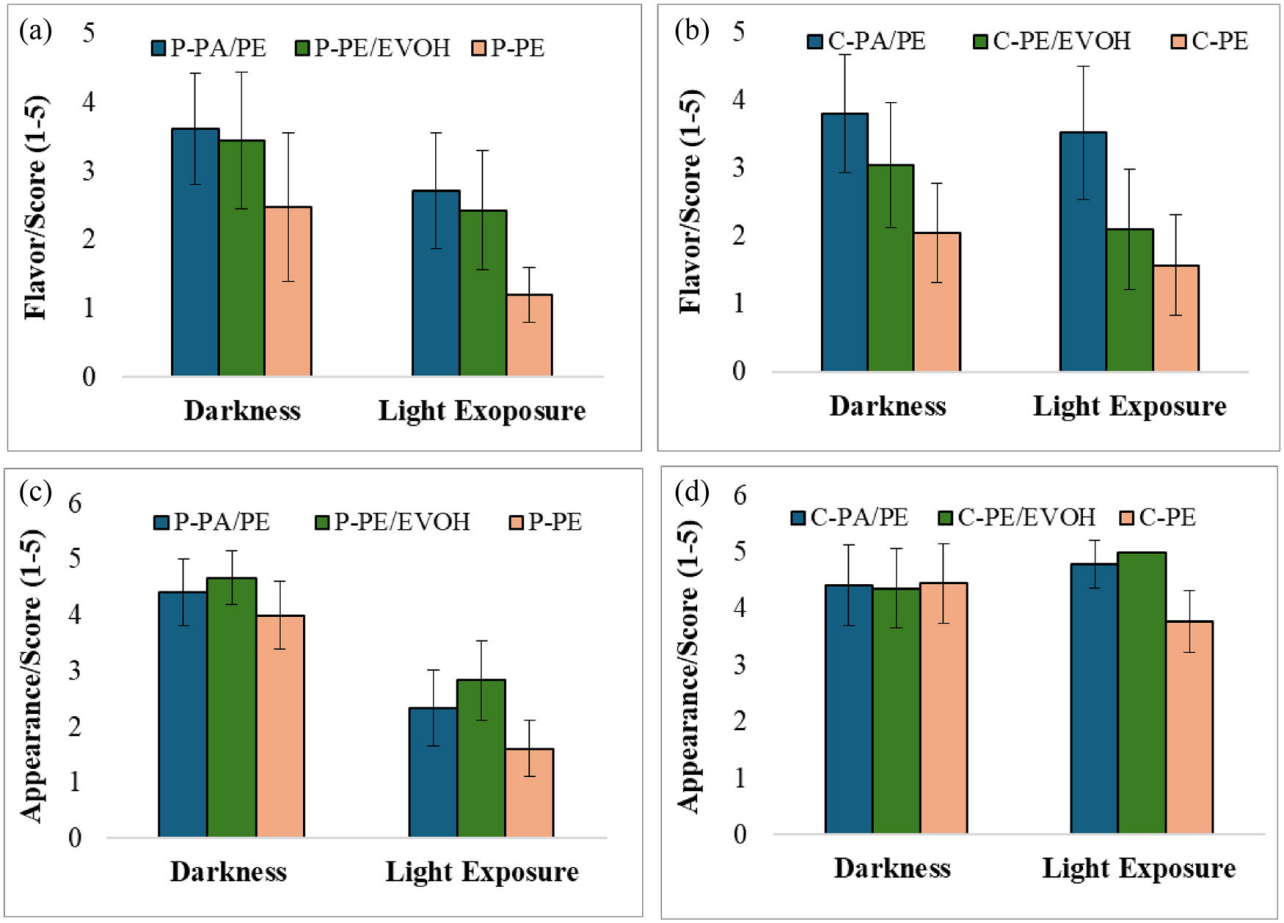
Flavor and appearance of mashed potato (a and c) and ground carrot (b and d) stored in both dark and light for 6 weeks. The values are mean of four measurements, and the error bars represent the standard deviation. EVOH, ethylene vinyl alcohol; PA, polyamides; PE, polyethylene.

Analogous results were observed for the flavor when stored in PE, and a significant reduction from 2.5 to 1.2 in dark and light was found, respectively (Figure 4). However, light exposure elicited a more pronounced and significant reduction in flavor in PE/EVOH (3.5–2.4) and PA/PE (3.6–2.7) compared to the reduction in odor. Storage in PE caused significant lower score compared to storage in PA/PE and PE/EVOH in dark as well as after light exposure. No significant differences between storage in PE/PE and PE/EVOH in the dark nor in light could be observed. According to the GLM packaging, both factors (packaging and condition) were significant for odor and flavor (packaging explained 28.3% and 25.6% and condition 17.1 and 21.5% of the variation in odor and flavor, respectively. *R* ^2^ adj. were 46.8% and 45.2%). For the odor, the interaction was also significant, although it only explained only 3.7% of the variation (as shown in Figure 4 and Table 2a).

As described, the odor and flavor of mashed potato were affected by light exposure and most notably when stored in low barrier material, PE. However, light exposure resulted in a pervasive and significant reduction in the appearance score for all samples. The reduction was more pronounced in PE and PA/PE (2.4 reduction) and 1.9 in the high barrier material PE/EVOH (PE 4.0–1.6, PE/EVOH 4.7–2.8, and PA/PE 4.7–2.3) (Figure 4). Similar results were observed in the color measurements with the largest reduction in hue/largest value in the total color difference.

These results are in line with the findings by Pettersen et al. (2022) for sous‐vide potato in which light exposure had a preponderant effect on the appearance compared to odor and flavor. They compared PE/EVOH (170 µm) and PP/EVOH material with PA/EVOH/PE (150 µm). Light exposure had more effect on the color and appearance of the potatoes when stored in PP/EVOH and PE/EVOH compared to the potatoes stored in PA/EVOH/PE; however, there was no effect on odor and flavor. These differences may in part be explained by the product characteristics sous‐vide potato versus mashed potatoes (instant potato mash with milk and containing oil), and the material thickness (PE/EVOH 170 and 150 µm in the present study) and moreover the structure (PA/EVOH/PE 150 µm vs. PA/PE 90 µm in the present study).

Light exposure had a significant effect on the odor (Table 5) and flavor of the ground carrot stored only in PE/EVOH (3.3–2.6 in odor and 3.0–2.1 in flavor) (Figure 4b), in contrast to the potatoes where light exposure elicited a reduction in odor and flavor in all materials. Significant alteration in odor and flavor was observed for carrot in PE stored for 6 weeks in darkness, and already regarded as not acceptable which can be explained by the barrier properties. Additionally, light did not entail further reduction in odor and only slight reduction in flavor (2.0–1.6). Packaging significantly affected the odor and flavor according to the GLM (packaging explained 36.1% and 41.8% of the variation), while storage condition (light) had a significant effect only on the flavor (explained 5.8%, *R* ^2^ adj. 36.6% and 46.9% for odor and flavor, respectively) (Table 2a).

Light exposure resulted in unacceptable odor and flavor for ground carrot stored in PE/EVOH. This is in conflict with what could be expected based on the OTR of these materials. The OTR of PE/EVOH increased due to heat treatment (0.86) and further increased due to food contact (0.93 carrot/0.97 potato) conversely to PA/PE (Table 2). However, the OTR of PA/PE still remains significantly higher than PE/EVOH.

The appearance of the carrot was in general not negatively affected by the light exposure (with a slight increase in score for PE/EVOH and PA/PE; Figure 4d), in clear distinction to the effect for potato. No significant differences were observed for samples stored in dark, while ground carrot stored in PE had a significant lower score compared to storage in the barrier materials PA/PE and PE/EVOH after light exposure (Table 5). It should be noted that bags with carrot only contained ground carrot without any additives, while the mashed potato was based on instant potato mash with milk, and containing oil. Furthermore, the inherent properties and characteristics of carrot and potato are different. For instance, fresh carrot and fresh potato are differently affected by light exposure, as fresh potatoes turn green due to chlorophyll development, and for cooked potato, photo‐oxidation of ferri‐chlorogenic acid is responsible for color changes, while carrot is unaffected by light (Wang‐Pruski & Nowak, 2004).

##### 3.2.2.3 Microbial levels

Samples subjected to light exposure were analyzed at week 6. No significant effects (*p *> 0.061) of neither light nor packaging material were found on any of the packaged products regarding microbial growth. In addition, no significant (*p* > 0.175) interactions were detected.

## 4 CONCLUSION

The PE/EVOH film had the lowest OTR by far, even after food contact and heat processing, and a WVTR comparable to the other two films. PE has a high OTR; food contact increased OTR, while heat treatment reduced it for PE. The tensile strength of all films was only reduced up to 3–4 weeks of food contact. Contact with food did not have any effect on the elongation at break of PE/EVOH films, while for PA/ PE and PE films, there was a reduction in the elongation at break after 3 weeks. With further contact up to 12 weeks, however, the films regained their initial elongation at break. For instrumentally measured color, no significant differences were seen in hue values for mashed potato between the different packaging materials during the first 6 weeks of dark storage. After 10 weeks, PE‐film showed significantly lower hue values and larger total color difference than the two other films. Light exposure reduced the hue values and increased total color difference for all packaging films after 6 weeks of storage. PE showed significantly lower hue values and higher total color difference, indicating that light, in combination with high OTR, increased the oxidation rate of the pigments in mashed potato.

Mashed potato showed a slight reduction in freshness‐odor for all materials with storage time, and when packaged in PE showed slightly lower freshness‐score compared to packaging in materials with higher oxygen barrier. The flavor of mashed potato was also scored lower when packaged in PE compared to both PE/EVOH and PA/PE, and the differences correspond well to the OTR of the materials. The low barrier PE resulted in unacceptable flavor in mashed potato after 6 weeks storage compared to after 10 weeks storage in high barrier materials. Similar trends were seen for ground carrot which, when packaged in the low barrier PE, was regarded unacceptable after 6 weeks. But when packaged in high barrier materials ground carrot was still acceptable after 10 weeks of storage. The odor and flavor of mashed potato were affected by light exposure and most notably when stored in low barrier material. However, light exposure resulted in pervasive and significant reduction in the appearance score for mashed potatoes. For ground carrot, light exposure had a significant effect on the odor and flavor, while no effect was observed on the color. Our results demonstrate the importance of barrier properties for the shelf life of the investigated vegetable model products and indicate that the introduction of a barrier layer in a recyclable film tends to increase the product shelf life to an extent that can be compared to non‐recyclable materials.

## AUTHOR CONTRIBUTIONS

**Nusrat Sharmin**: Investigation; formal analysis; data curation; writing—review and editing; visualization; writing—original draft; validation. **Bjørn Tore Rotabakk**: Conceptualization; methodology; data curation; formal analysis; validation; investigation; visualization; writing—review and editing; writing—original draft; resources. **Magnhild Seim Grøvlen**: Methodology; data curation; investigation; writing—review and editing; formal analysis; resources. **Hanne Larsen**: Conceptualization; validation; data curation; formal analysis; writing—original draft; writing—review and editing. **Torstein Skåra**: Methodology; writing—original draft; writing—review and editing; data curation; formal analysis. **Marit Kvalvåg Pettersen**: Conceptualization; methodology; data curation; supervision; formal analysis; project administration; validation; visualization; investigation; funding acquisition; writing—original draft; writing—review and editing.

## CONFLICT OF INTEREST STATEMENT

The authors have no conflict of interest to declare.

## Supporting information



Table S1: Summaries of analyses of variance for color measurements on mashed potato and ground carrot after 6 weeks of storage both in dark and light. Numbers show percentage of variation per factor/interaction. Table shows % percentage of variation per factor/interaction. and p‐values (significant (p values < 0.05) factors are marked green)
Table S2: Summaries of analyses of variance for measurements on mashed potato and ground carrot. Table shows % percentage of variation per factor/interaction. and p‐values (significant (p values < 0.05) factors are marked green)
Table S3: Summaries of analyses of variance for odor, flavor and appearance of mashed potato and ground carrot after 3–10 weeks of storage in dark. Numbers show percentage of variation per factor/interaction. Table shows % percentage of variation per factor/interaction. and p‐values (significant  (p values < 0.05)  factors are marked green)
Figure S1: Mashed potato stored in PA/PE, PE and PE/EVOH for 6 weeks.
