# Supplementary material for: Recyclable and non‐recyclable packaging films with different barrier properties: Effect of processing and storage time on quality of mashed potato and ground carrot
Source: J Food Sci. 2024 Dec 1;89(12):9466–82. doi: 10.1111/1750-3841.17486 (PMC11673516; doi:10.1111/1750-3841.17486)
Supplement: Supplementary file 1 — Table S1: Summaries of analyses of variance for color measurements on mashed potato and ground carrot after 6 weeks of storage both in dark and light. Numbers show percentage of variation per factor/interaction. Table shows % percentage of variation per factor/interaction. and p‐values (significant (p values < 0.05) factors are marked green) Table S2: Summaries of analyses of variance for measurements on mashed potato and ground carrot. Table shows % percentage of variation per factor/interaction. and p‐values (significant (p values < 0.05) factors are marked green) Table S3: Summaries of analyses of variance for odor, flavor and appearance of mashed potato and ground carrot after 3–10 weeks of storage in dark. Numbers show percentage of variation per factor/interaction. Table shows % percentage of variation per factor/interaction. and p‐values (significant (p values < 0.05) factors are marked green) Figure S1: Mashed potato stored in PA/PE, PE and PE/EVOH for 6 weeks. [file JFDS-89-9466-s001.docx]

Table S1: Summaries of analyses of variance for color measurements on mashed potato and ground carrot after 6 weeks of storage both in dark and light. Numbers show percentage of variation per factor/interaction. Table shows % percentage of variation per factor/interaction. and p-values (significant  (p values < 0.05)  factors are marked green)

| 6 weeks storage dark/light. Method Packaging (P), Storage condition (C), P*C | | | | | | | | |
| --- | --- | --- | --- | --- | --- | --- | --- | --- |
|  | Mashed potato | | | | | | | |
|  | L* | | C* | | h° | | ∆E | |
| Factors/interaction | % variation | p-value | % variation | p-value | % variation | p-value | % variation | p-value |
| Packaging (P) | 9,57 | 0,024 | 0,17 | 0,483 | 13,46 | 0,001 | 12,89 | 0,000 |
| Storage condition (C) | 47,58 | 0,000 | 95,98 | 0,000 | 55,35 | 0,000 | 68,64 | 0,000 |
| Interaction (P*C) | 8,92 | 0,030 | 0,40 | 0,194 | 9,36 | 0,005 | 4,11 | 0,023 |
| Error | 33,94 |  | 3,45 |  | 21,83 |  | 14,35 |  |
| R-Sq(adj) | 60,41 |  | 95,97 |  | 74,53 |  | 83,25 |  |
| 6 weeks storage dark/light. Method Packaging (P), Storage condition (C), P*C | | | | | | | | |
|  | Ground carrot | | | | | | | |
|  | L* | | C* | | h° | | ∆E | |
| Factors/interaction | % variation | p-value | % variation | p-value | % variation | p-value | % variation | p-value |
| Packaging (P) | 9,65 | 0,160 | 23,49 | 0,011 | 1,54 | 0,650 | 62,76 | 0,000 |
| Storage conditon (C) | 9,56 | 0,059 | 2,60 | 0,291 | 41,46 | 0,000 | 3,54 | 0,080 |
| Interaction (P*C) | 6,54 | 0,282 | 6,35 | 0,260 | 3,96 | 0,339 | 1,38 | 0,534 |
| Error | 74,24 |  | 67,56 |  | 53,03 |  | 32,31 |  |
| R-Sq(adj) | 13,38 |  | 21,18 |  | 38,13 |  | 62,30 |  |

Table S2: Summaries of analyses of variance for measurements on mashed potato and ground carrot. Table shows % percentage of variation per factor/interaction. and p-values (significant  (p values < 0.05)  factors are marked green)

| 6 weeks storage dark/light. Method: Packaging (P) , Storage condition (C), P*C | | | | | |  |
| --- | --- | --- | --- | --- | --- | --- |
| Mashed Potato | | | | | | |
|  | Odor |  | Flavor |  | Appearance |  |
| Factors/interaction | % variation | *p-value* | % variation | *p-value* | % variation | *p-value* |
| Packaging (P) | 28,31 | *0,0000* | 25,57 | *0,0000* | 9,19 | *0,000* |
| Storage Condition (C) | 17,10 | *0,0000* | 21,47 | *0,0000* | 69,31 | *0,000* |
| P*C | 3,70 | *0,0160* | 0,48 | *0,5830* | 0,91 | *0,106* |
| Error | 51,02 |  | 52,64 |  | 19,97 |  |
| Total | 100,13 |  | 100,16 |  | 99,38 |  |
| R-Sq-adj | 46,83 % |  | 45,15 % |  | 79,05 % |  |
|  |  |  |  |  |  |  |
| Ground Carrot | | | | | | |
|  | Odour |  | Flavour |  | Appearance |  |
| Factors/interaction | % variation | *p-value* | % variation | *p-value* | % variation | *p-value* |
| Packaging (P) | 36,05 | *0,0000* | 41,76 | *0,0000* | 41,76 | *0,000* |
| Storage Condition (C) | 1,15 | *0,1350* | 5,87 | *0,0000* | 5,87 | *0,000* |
| P*C | 1,95 | *0,1510* | 1,41 | *0,1940* | 1,41 | *0,194* |
| Error | 60,85 |  | 50,96 |  | 50,96 |  |
| Total | 100,00 |  | 100,00 |  | 100,00 |  |
| R-Sq-adj | 36,61 % |  | 46,92 % |  | 46,92 % |  |

Table S3: Summaries of analyses of variance for odor, flavor and appearance of mashed potato and ground carrot after 3-10 weeks of storage in dark. Numbers show percentage of variation per factor/interaction. Table shows % percentage of variation per factor/interaction. and p-values (significant  (p values < 0.05)  factors are marked green)

| 3-10 weeks storage dark. Method: Packaging (P) , Storage condition (C), P*C | | | | | | |
| --- | --- | --- | --- | --- | --- | --- |
| Mashed Potato | | | | | | |
|  | Odour |  | Flavour |  | Appearance |  |
| Factors/interaction | % variation | *p-value* | % variation | *p-value* | % variation | *p-value* |
| Packaging (P) | 4,21 | *0,018* | 8,98 | *0,000* | 25,51 | *0,000* |
| Storage Condition (C) | 5,36 | *0,006* | 14,48 | *0,000* | 23,99 | *0,000* |
| P*C | 7,46 | *0,007* | 7,45 | *0,002* | 10,42 | *0,000* |
| Error | 82,31 |  | 68,37 |  | 37,43 |  |
| Total | 99,34 |  | 99,28 |  | 97,36 |  |
| R-Sq-adj | 13,60% |  | 28,23% |  | 60,46% |  |
|  |  |  |  |  |  |  |
| Ground Carrot | | | | | | |
|  | Odour |  | Flavour |  | Appearance |  |
| Factors/interaction | % variation | *p-value* | % variation | *p-value* | % variation | *p-value* |
| Packaging (P) | 21,24 | *0,000* | 20,09 | *0,000* | 15,13 | *0,000* |
| Storage Condition (C) | 24,14 | *0,000* | 26,25 | *0,000* | 4,13 | *0,024* |
| P*C | 6,02 | *0,001* | 3,94 | *0,013* | 10,05 | *0,001* |
| Error | 47,32 |  | 48,51 |  | 71,08 |  |
| Total | 98,72 |  | 98,81 |  | 100,40 |  |
| R-Sq-adj | 50,33% |  | 49,08% |  | 24,61% |  |


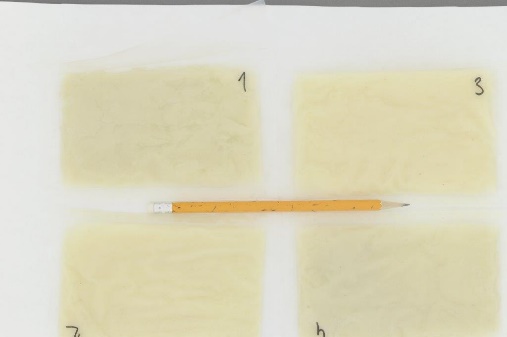

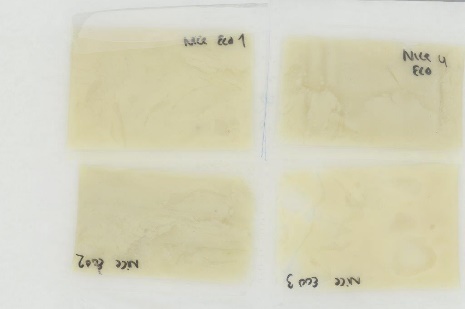

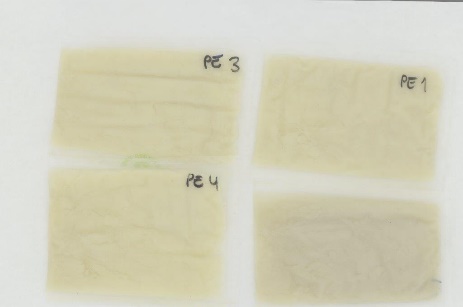


PA/PE 6 weeks

PE 6 weeks

PE/EVOH 6 weeks

Figure S1: Mashed potato stored in PA/PE, PE and PE/EVOH for 6 weeks.


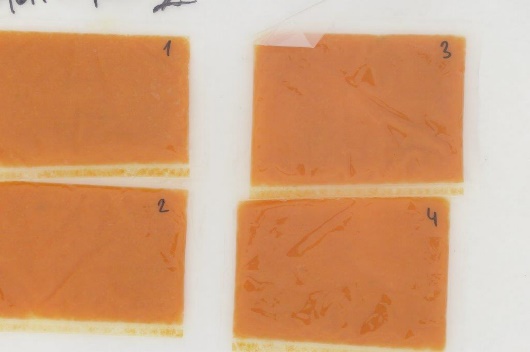

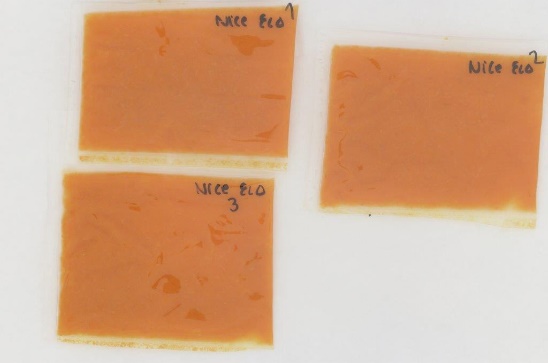

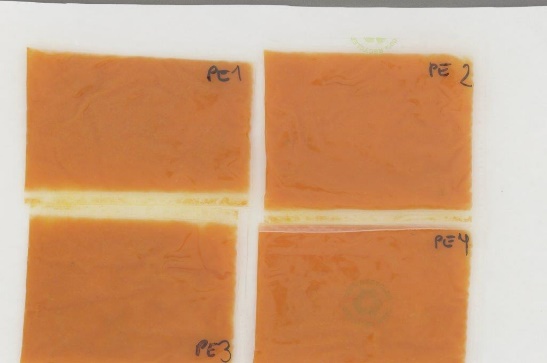


PA/PE 6 weeks

PE/EVOH 6 weeks

PE 6 weeks

Figure S1: Ground carrot stored in PA/PE, PE and PE/EVOH for 6 weeks.
